# Sexual health indicators for the United States: Measuring progress and documenting public health needs

**DOI:** 10.3389/fpubh.2022.1040097

**Published:** 2023-01-26

**Authors:** Jessie V. Ford, Megan B. Ivankovich, Eli Coleman

**Affiliations:** ^1^Department of Sociomedical Sciences, Columbia University, New York, NY, United States; ^2^Population Reference Bureau, Washington, DC, United States; ^3^Institute for Sexual and Gender Health, University of Minnesota Twin Cities, St. Paul, MN, United States

**Keywords:** sexual health, sexual wellbeing, sexually transmitted infections, reproductive health, indicators and metrics

## Abstract

**Introduction:**

Today, we are facing increased and continued adverse sexual health outcomes in the United States, including high post-COVID-19 pandemic rates of sexually transmitted infections (STIs). For the past 20 years, there have been calls for a national health strategy and a more comprehensive sexual health approach to address the myriad of persistent sexual health problems in this country. Employing a sexual health approach requires shifting from a longstanding, stigmatizing focus on morbidity toward a holistic and integrated focus on health rather than disease. While strategies are being implemented by multisectoral stakeholders, it is also important to establish a core set of indicators that broadly describe the state of sexual health in the U.S. and allow for measurement across time. The development of a comprehensive scorecard with key sexual health indicators has been proposed by other entities (e.g., Public Health England, World Health Organization), but such an attempt has not been made in the U.S.

**Methods:**

A review of national U.S. surveys and surveillance systems with items related to sexual health was conducted for years 2010–2022 to develop an inventory of existing data that yield national estimates for potential indicators of sexual health.

**Results:**

We selected 23 sexual health indicators in four broad domains including: (1) knowledge; communication and attitudes (five indicators); (2) behaviors and relationships (four indicators); (3) service access and utilization (seven indicators); and (4) adverse health outcomes (seven indicators). Recent data for each indicator are provided.

**Discussion:**

A growing body of evidence shows the positive effects of moving away from a morbidity focus toward an integrated, health-promoting approach to sexual health. Yet, not much has been done in terms of how we implement this national shift. We argue that measurement and monitoring are key to future change. We envision these core sexual health indicators would be published in the form of an index that is publicly available and updated frequently. These sexual health indicators could be used for ongoing monitoring, and to guide related research, programming, and policy development to help promote sexual health in coming years.

## Introduction

During the COVID-19 pandemic, rates of sexually transmitted infections (STIs) increased substantially. This trend—driven by delays in surveillance and treatment—reminds us that sexual health matters even in the face of a pandemic (1–3). Amidst the recent overturning of Roe v. Wade we are reminded again that sexual health has profound impacts on people's lives. For the last 20 years, there have been calls for a more holistic and integrated approach to sexual health in the U.S. (3–8). Such an approach would help combat persistent public health challenges, including HIV, STIs, unintended pregnancy, and sexual violence. “Sexual health” is defined by the World Health Organization (WHO) as “a state of physical, emotional, mental and social wellbeing in relation to sexuality; it is not merely the absence of disease, dysfunction or infirmity. Sexual health requires a positive and respectful approach to sexuality and sexual relationships, as well as the possibility of having pleasurable and safe sexual experiences, free of coercion, discrimination, and violence.” (9). Employing a sexual health approach involves shifting away from an enduring, stigmatizing focus on morbidity toward a focus on health rather than disease across the lifecourse (3, 10–12). In turn, sexual health services and funding would be less fragmented, costs lowered, stigma decreased, and health and productivity improved (3, 7, 10–14).

While we have not yet succeeded in generating a broadly integrated national approach to sexual health in the U.S., some essential efforts are already in place. For example, our National HIV/AIDS Strategy (2022–2025) (15), STI Treatment Guidelines (2021) (1), and the Center for Disease Control and Prevention (CDC's) new unintended pregnancy prevention initiative (16) have all recently been updated with a renewed focus on overall health. Yet, there is room for more progress. In this article, we present a baseline set of sexual health indicators designed as a foundation upon which to build our future sexual health.

## Background

Current data provide evidence of persistent sub-optimal sexual health in the U.S. STIs such as chlamydia and gonorrhea are the most commonly reported infectious diseases and HIV and syphilis consistently rank highly on the list of notifiable diseases compiled annually by the Centers for Disease Control and Prevention (CDC) (17). Almost half of all pregnancies are unintended (18) and sexual violence is common (19). Sexual dysfunction (e.g., erectile dysfunction, painful intercourse) is reported by at least half of older Americans (20). Reproductive tract cancers affect hundreds of thousands of people annually (21).

Many of these outcomes occur in the same individuals and subpopulations, creating syndemics (overlapping epidemics of two or more health-related issues), furthering the burden of disease (22). Syndemics most commonly emerge under conditions of health inequality created by poverty, stigma, or structural violence where these factors work together to heighten vulnerability (23). HIV and viral hepatitis, for example, form a syndemic affecting vulnerable populations namely people who use drugs. Despite the accessibility of treatments, this syndemic has become of increasing concern in U.S. areas most affected by the social drivers of opioids and other substance use (24).

Adverse sexual health outcomes are not just too common, they are also costly, placing a large burden on health care systems (3, 10). The direct medical costs of STIs including sexually transmitted HIV and hepatitis B are estimated at $16 billion dollars annually (22, 25). Similarly, the social and economic impact of unintended and teen pregnancy in the U.S. is substantial, with $9 billion annually in estimated costs related to teen pregnancy alone (26). Rape and other forms of sexual assault are estimated to cause an annual minimum loss of $12 billion (27, 28); these estimates do not account for the emotional and psychological burdens of such acts.

Historically, public health efforts to address adverse sexual health outcomes have typically focused on a single outcome or disease rather than examining sexual health in a holistic and integrated manner. For example, many public health programs, such as family planning or STI/HIV prevention, are funded based on the topic area, with organizational separation (7, 10). To address this tendency toward such isolated efforts, there have been calls for a national health strategy and a more comprehensive sexual health approach to address the myriad of persistent sexual health problems. Beginning in 2001, *The Surgeon General's Call to Action to Promote Sexual Health and Responsible Sexual Behavior* was the first formal U.S. government recognition of the need to broadly promote sexual health to enhance overall population health (4). More recently, Healthy People 2030, the White House National Strategy on Gender Equity and Equality, and the World Health Organization's Global STI Strategy all echo this focus on sexual health, emphasizing health, wellbeing, prevention, and relationships (1, 6, 13, 29). The Department of Health and Human Services (HHS) recently developed a blueprint for how to include sexual health across all HHS programs, including Medicare and Medicaid (30). Finally, the National Academies of Sciences, Engineering, and Medicine (NASEM) called for moving from a narrow view of STI prevention to a broader, holistic sexual health approach that addresses the social and structural determinants of sexual behavior (7).

In dialogue with these national efforts, a novel model for action was proposed in 2013 (11) and again in 2017 (3) for public health partners to promote sexual health across the lifespan This framework—based upon an emphasis on wellness, focus on positive and respectful relationships, acknowledgment of sexual health as a component of overall health, and integrated approach to prevention—includes four primary long-term objectives: (1) Increase knowledge, communication, and respectful attitudes; (2) Increase healthy, responsible, and respectful sexual behaviors and relationships; (3) Increase use of high quality, coordinated educational, clinical, and other preventive services; and (4) Decrease adverse sexual health outcomes (detailed below) (11). To support these long-term sexual health objectives, it is essential to establish a core set of indicators that broadly describe the state of sexual health in the U.S. and allow for tracking across time. Collecting better measures of sexual health will not only help address recent rises in STI rates, it will help efforts to manage other syndemics such as HIV, substance use, and mental health disorders.

Notably, the development of a comprehensive scorecard with key sexual health indicators has been proposed by some entities [e.g., Public Health England, United Nations Population Fund (UNFPA), WHO] (31–33). Likewise, a growing number of countries have conducted national sexual health surveys (e.g., Australia, Canada, Flanders, France, Germany, Ireland, Latvia, Malta, The Netherlands, Portugal, Spain, and the United Kingdom) (34–37). We have not seen efforts to this extent in the U.S. At present, these countries and organizations use this type of data to monitor the sexual health of their population, to support local and national efforts, and to determine the impact of public health related systems (32, 38). We anticipate that a proposed U.S. scorecard might be used in a similar way (e.g., for monitoring, to inform related research, programming, and policy development). We elaborate on this further in the discussion.

Accordingly, we reviewed existing data systems that yield national estimates for potential indicators of sexual health (8). Although numerous high-quality data sources exist, none provided a comprehensive assessment of sexual health across the lifespan and included the domains of knowledge, communication, attitudes, service access and utilization, behaviors, relationships, and adverse health outcomes (8). Therefore, we sought to identify a core set of measures from multiple existing data systems and to characterize the current state of sexual health in the U.S.

## Methods

Two sets of meetings were held to review and identify a core set of sexual health indicators. First, representatives from six CDC divisions addressing aspects of sexual health (i.e., HIV/AIDS, STD, viral hepatitis, adolescent/school health, reproductive health, sexual violence) met in 2010–2011 to review and identify a core set of sexual health indicators. Subject matter experts proposed indicators based on their understanding of their respective fields' best available measurements; all indicators were selected through a consensus decision-making process. These indicators were shared with external partners active in the CDC/Health Resources and Services Administration (HRSA) Advisory Committee's sexual health workgroup. Recognizing the critical state of sexual health during the COVID-19 pandemic, efforts to finalize indicator selection and dissemination continued: a second meeting was held in 2022 with public health representatives, including academics, non-governmental organizations, and CDC experts, to reexamine the indicators considering the current state of public health. Based off feedback provided at this meeting, we: (1) confirmed the indicators corresponded with Healthy People 2030 targets (rather than Healthy People 2020); (2) made sure the indicators aligned with 2021 CDC STI treatment guidelines and NASEM report both calling for attention to sexual health and reduction of stigma; and (3) changed one indicator measuring gonorrhea to syphilis based on current guidelines (1).

Indicators were selected to measure long-term sexual health outcomes (3, 11) and to allow for future tracking (ideally, every 1–2 years). Additionally, selection criteria included: (1) identifying a concise set of indicators; (2) focusing on the general population; (3) identifying indicators that would link policy and action; and (4) aligning these measures with existing targets or goals (e.g., Healthy People 2030).

We established a target range for number of indicators (18–23 total) for the aforementioned four primary long-term objectives for improving population sexual health: (1) Increase knowledge, communication, and respectful attitudes (3–4 indicators); (2) Increase healthy, responsible, and respectful sexual behaviors and relationships (3–4 indicators); (3) Increase use of high quality, coordinated educational, clinical, and other preventive services that improve sexual health (6–7 indicators); and (4) Decrease adverse health outcomes, including HIV/STIs, viral hepatitis, unintended pregnancies, sexual violence, sexual dysfunction, and cancers in reproductive tracts (6–7 indicators). Within each objective, we attempted to select at least one indicator per domain (domains summarized in Table 1). For example, for Objective 1, we identified at least one indicator for each domain: knowledge, communication, and attitudes. We thought 18–23 indicators could capture the range of domains covered and topics represented in sexual health while being a manageable set to track over time.

**Table 1:** Summary of sexual health indicators.

	**CDC surveillance**	**GSS**	**HEDIS**	**NAACCR**	**NIS-Teen**	**NISVS**	**NSFG**	**NVSS**	**Profiles**
**1. Knowledge, communication, attitudes**									
Formal sex education							X		
Communication with parents							X		
Condom communication							X		
Attitudes toward same-sex sex		X							
Presence of gay/straight alliance									X
**2. Behaviors and relationships**									
Sexual debut							X		
Condom use							X		
Contraceptive use							X		
Relationship happiness		X							
**3. Education and services**									
Sexual health course required									X
Bullying and harassment									X
Receipt of health services							X		
HIV testing	X								
Chlamydia screening			X						
HPV vaccine					X				
Prenatal care								X	
**4. Adverse outcomes**									
HIV	X								
Syphilis	X								
Hepatitis B	X								
Unintended pregnancy							X	X	
Teen pregnancy							X	X	
Sexual violence						X			
Reproductive cancer				X					

Our primary source in this process was an existing review study that identified 18 U.S.-focused nationally-representative surveys and surveillance systems that provided individual-level data, addressed elements of sexual health, and had a high likelihood of future data collection (8). We also selected indicators that best aligned with the strategic goals such as those included in Healthy People 2030 and the National Strategy on Gender Equity and Equality. When a sufficient indicator at the individual level was not available, we considered national-level data at other levels (e.g., institutional).

## Results

The selected indicators of sexual health in the U.S. are described below and summarized in Table 1. We provide further information on indicators' data sources, and measurement notes including a detailed description of indicator components in Table 2. We provide recent data for each indicator in Table 3.

**Table 2:** Selected sexual health indicators by objectives.

**Sexual health indicator**	**Data source**	**Population**	**Alignment with existing published goals/objectives**	**Measurement notes^a^**
**Objective 1. Increase knowledge, communication, and respectful attitudes regarding sexual health**				
**Knowledge**				
1.A. Percentage of youth that received formal sex education before they were 18 years old	NSFG	M&F, 15–24 yrs	HP2030 (FP-08) -includes separate measures for 4 topics; 15–19 yrs	Composite indicator, requiring “yes” response to all 4 topics: saying no to sex; methods of contraception; STIs; HIV/AIDS prevention (2–3 years)
**Communication**				
1.B. Percentage of adolescents that have talked with a parent or guardian about several sexual health topics	NSFG	M&F, 15–19 yrs	HP2020 (FP-13)	Composite indicator, requiring “yes” response to all 4 topics: how to say no to sex; methods of contraception; STIs; how to use a condom (2–3 years)
1.C. Percentage of youth that report it would not be embarrassing to discuss using a condom with a new partner	NSFG	M&F, 15–24 yrs	–	Represents youth who report there is ‘no chance' a condom discussion would be embarrassing (2–3 years)
**Attitudes**				
1.D. Percentage of people that agree sexual relations between two adults of the same sex are not wrong	GSS	M&F, >17 yrs	–	Combines two categories: “wrong only sometimes” and “not wrong at all” (biennial)
1. E. Proportion of middle and high schools with gay-straight alliances (GSA)	School Health Profiles (Profiles)	M&F middle and high school youth	–	Based upon response that “yes” school has a GSA or similar club (biennial)
**Objective 2. Increase healthy, responsible, and respectful sexual behaviors and relationships**				
**Sexual behaviors**				
2.A. Percentage of adolescents who never had sexual intercourse (aged 15 yrs)	NSFG	M&F, 15 yrs	HP2030 (FP-04)	Sexual intercourse = vaginal sex. Slight wording change from HP2030. (2–3 years)
2.B. Percentage of sexually active unmarried persons who use condoms	NSFG	M&F, 15–44 yrs	HP2030 (FP-05; FP-06)	Based upon last vaginal sex (2–3 years)
2.C. Percentage of percent of women aged 20 to 44 years at risk of unintended pregnancy who used most effective or moderately effective methods of contraception	NSFG	F, 15–44 yrs	HP2030 (FP-10)	Based upon last vaginal sex (2–3 years)
**Relationships**				
2.D. Percentage of people that report happiness with marriage	GSS	M&F, >17 yrs	–	Represents adults who selected “very happy” (biennial)
**Objective 3. Increase use of high-quality, coordinated educational, clinical, and other preventive services that improve sexual health**				
3.A. Percentage of schools teaching 20 key HIV, STI, and pregnancy prevention topics in a required course	School Health Profiles	Middle & high schools	–	Composite indicators, requiring “yes” response to 20 age-appropriate sexual health topics, by grade level
3.B. Percentage of schools with practices in place to prevent bullying and sexual harassment	School Health Profiles	Middle & high schools	–	Composite performance indicator requiring “yes” response to 4 key practices in place to prevent bullying and sexual harassment
3.C. Percentage of sexually active persons who receive sexual and reproductive health services	NSFG	M&F, 15–44 yrs	HP2020 (FP-7), NPS	Sexually experienced men and women who received ≥ 1 reproductive health services in the past 12 months^b^
3.D. Percentage of people living with HIV who know their serostatus	HIV surveillance	>12 yrs	HP2030 (HIV-02), NHAS, NPS	Estimates derived using extended back-calculation on HIV and acquired immunodeficiency syndrome (AIDS) data at diagnosis from 40 states (with confidential name-based HIV infection reporting since at least January 2006), and AIDS data from 10 states and the District of Columbia. (annual)
3.E. Proportion of sexually active adolescent and young females enrolled in Medicaid and commercial health plans who are screened for chlamydial infections	HEDIS	F, 16–24 yrs	HP2030 (STI-01), NPS, STI Plan	Participating health plans report the annual Chlamydia screening rate measured by the proportion of sexually active women (annual)
3.F. Percentage of adolescents aged 13 through 15 years who received recommended doses of the HPV vaccine	NIS-Teen	13–15 yrs	–	Vaccination coverage estimates are available for adolescents with adequate provider data
3.G. Percentage of pregnant women who receive early and adequate prenatal care	NVSS	F, 15–44 yrs	HP2030 (MICH-08)	Uses APNCU measure combining number and timing of prenatal visits (annual)
**Objective 4. Decrease adverse health outcomes, including HIV/STIs, viral hepatitis, unintended pregnancies, sexual violence, sexual dysfunction, and cancers in reproductive tracts**				
4.A. New (incident) HIV infections among adolescents and adults	HIV incidence surveillance	M&F, >12 yrs	HP2030 (HIV-1), NHAS	Incidence is statistically estimated by CDC (part of NNDSS) (annual)
4.B. Rate of syphilis in women (15–44 yrs) and MSM (all ages) (per 100,000)	STD surveillance	F, 15–44 yrs; all MSM	HP2030 (STI-03; STI-05), STI Plan	Nationally notifiable STD surveillance data (part of NNDSS) (annual)
4.C. New hepatitis B infections in adults	Viral hepatitis surveillance	M&F, >17 yrs	NNDSS; HP2030 (IID-11)	New infections are statistically estimated by CDC using reported cases (part of NNDSS) (annual)
4.D. Percentage of pregnancies that are unintended	NSFG, NVSS, Guttmacher, CDC Abortion Surveillance Data	F, 15–44 yrs	HP2030 (FP-1)	Modification of HP2020 indicator. Calculated using several data sources (4–5 years)
4.E. Pregnancy rates among adolescent females (per 1,000)	Abortion Provider Survey, Guttmacher Institute; NVSS; NSFG; Abortion Surveillance Data	F, 15–19 yrs	HP2030 (FP-3), NPS	Calculated using several data sources (annual)
4.F. Prevalence of sexual violence in past 12 months (by any perpetrator)	NISVS	M&F, >17 yrs	HP2030 (IVP-05)	Rape (completed and attempted) and unwanted sexual contact involving touch but not sexual penetration. (annual)
4.H Incidence of cancers of the reproductive tract	NAACCR	M&F, >17 yrs	–	Cancers associated with genital HPV in men and women; prostate cancer and cervical, ovarian, uterine, vaginal, and vulvar cancer

^a^Projected timeframe for data availability is provided in parentheses. ^b^Services included by sex were: Males (received advice/counseling regarding female birth control methods, male birth control methods, getting surgically sterilized, STIs, or HIV/AIDS); Females (received birth control method or counseling, birth control check-up/medical test, sterilization counseling, emergency contraception counseling, pelvic exam, pap smear, pregnancy test, or STI counseling/treatment).

**Table 3:** Recent data for sexual health indicators.

**Sexual health indicator**	**Year**	**Most recent data**
**Objective 1. Increase knowledge, communication, and respectful attitudes regarding sexual health**		
**Knowledge**		
1.A. Percentage of youth that received formal sex education before they were 18 years old		
Female	2015–2019	53%
Male	2015–2019	54%
**Communication**		
1.B. Percentage of adolescents that have talked with a parent or guardian about several sexual health topics		
Female	2011–15	54%
Male	2011–15	47%
1.C. Percentage of youth that report it would not be embarrassing to discuss using a condom with a new partner		
Female	2011–15	63%
Male	2011–15	61%
**Attitudes**		
1.D. Percentage of people that agree sexual relations between two adults of the same sex are not wrong		
Female	2021	64%
Male	2021	60%
1.E. Percentage of middle and high schools with gay-straight alliances	2018	37%
**Objective 2. Increase healthy, responsible, and respectful sexual behaviors and relationships Sexual behaviors**		
2.A. Percentage of adolescents age 15–17 who never had sexual intercourse		
Female	2017–19	72%
Male	2017–19	77%
2.B. Proportion of sexually active adolescent females who used effective birth control and adolescent males who used a condom at last intercourse		
Female	2017–19	22%
Male	2017–19	67%
2.C. Percent of women at risk of unintended pregnancy who used most effective or moderately effective methods of contraception	2017–19	62%
**Relationships**		
2.D. Percentage of people that report happiness with marriage (very happy)		
Female	2021	49%
Male	2021	51%
**Objective 3. Increase use of evidence-based education and clinical and other preventive services that improve sexual health Education services**		
3.A. Percentage of schools teaching 20 key HIV, STIs, and pregnancy prevention topics in a required course		
Grades 6-8	2018	18%
Grades 9-12	2018	43%
3.B. Percentage of middle and high schools with 4 key practices in place to prevent bullying and sexual harassment	2018	49%
**Clinical and other preventive services**		
3.C. Percentage of sexually active persons who receive reproductive health services		
Female	2011–15	78%
Male	2011–15	13%
3.D. Percentage of people living with HIV who know their serostatus	2017	86%
3.E. Percentage of sexually active women enrolled in Medicaid and commercial plans who are screened for chlamydia		
Female	2018	56%
3.F. Percentage of adolescents aged 13–15 years received recommended doses of the HPV vaccine		
Adolescents	2020	55%
3.G. Percentage of pregnant women who receive early and adequate prenatal care	2020	75%
**Objective 4. Decrease adverse health outcomes, including HIV/AIDS, STIs, viral hepatitis, unintended pregnancies, and sexual violence**		
4.A. New (incident) HIV infections among adolescents and adults	2017	37,000
4.B. Cases of primary and secondary syphilis in women and men who have sex with men (MSM) (per 100,000)		
Women	2017	5.1
MSM	2018	401.1
4.C. New hepatitis B infections in adults^a^	2016	20,900
4.D. Percentage of pregnancies that are unintended	2014	45%
4.E. Pregnancy rates among adolescent females (per 1,000)	2016	20.3
4.F. Prevalence of sexual violence in past 12 months (by any perpetrator)		
Rape (completed or attempted): Female	2016–2017	2%
Unwanted sexual contact other than rape: Female	2016–2017	5%
Rape (completed or attempted): Male	2016–2017	< 1%
Unwanted sexual contact other than rape: Male	2016–2017	3%
4.G Estimated Cases of Genital System Cancers		
Female	2018	110,070
Male	2018	176,320

a Cases adjusted for underreporting.

### Objective 1. Increase knowledge, communication, and respectful attitudes regarding sexual health

Knowledge of sexual health is a complex concept that is not easily represented by the few existing items on national data sources (8). Therefore, although it is not comprehensive and may not adequately capture what youth have retained, we selected “youth (15–24 years) exposure to formal sex education (four topics) before age 18 years” (6, 39) for our knowledge indicator (Table 2, 1.A.). This indicator comes from the National Survey of Family Growth (NSFG) (40). In 2015–2019, ~54% of males and 53% of females reported receiving formal sex education on delaying sex, birth control methods, HIV/AIDS prevention, and sexually transmitted diseases before they were 18 years old (Table 3) (6, 41).

We also found measures of communication with partners, parents/family, and providers to be lacking in the existing data sources meeting our criteria. We selected the following as best available measures of parent and partner communication as sexual health indicators from the NSFG: “youth (15–19 years) who had talked with a parent or guardian about (four key) sexual health topics” (Table 2, 1.B.) and “who reported it would not be embarrassing to discuss using a condom with a new partner” (Table 2, 1.C.). NSFG data (2011–2015) show that 54% of females and 47% of males have talked with a parent or guardian about several sexual health topics (Table 3) (42). In addition, in 2011–2015, over 60% of youth reported that they would not be embarrassed to discuss condom use with a new partner (43).

For attitudes related to sexual health, we identified several measures that could serve as potential indicators; however, it was challenging to find measures of “respectful” attitudes given concerns about value bias or misinterpretation. Thus, we decided to focus on attitudes related to sexual health that could reduce the likelihood of discrimination. CDC states that “stigma and homophobia may have a profound impact on the lives of MSM [men who have sex with men], especially their mental and sexual health” (44); therefore, we selected the following attitudinal indicator from the General Social Survey (GSS): “What about sexual relations between adults of the same sex—do you think it is always wrong, almost always wrong, wrong only sometimes, or not wrong at all” (Table 2, 1.D) (45). In 2021, ~64% of females and 60% of males agreed that same-sex relations between adults were not wrong. Additionally, an indicator was also chosen from the CDC School Health Profiles (46) to measure attitudes toward sexual minority youth: the percentage of middle and high schools with gay-straight alliances (GSAs) (Table 2, 1.E). In 2018, 36.8% of secondary schools in the United States had a GSA or similar club (46). Research shows sexual minority youth who attend schools with gay/straight alliances are less likely than sexual minority youth who attend other schools to report dating violence, harassment or skipping school because they felt unsafe (47).

### Objective 2. Increase healthy, responsible, and respectful sexual behaviors and relationships

Three indicators were selected to assess healthy, responsible, and respectful sexual behaviors. As a measure of delay in initiation of sex, from NSFG we chose “adolescents aged 15–17 who had never had sexual intercourse (vaginal sex)” (Table 2, 2.A) (6). In 2017–2019, ~77% of male and 72% of female adolescents reported that they never had sexual intercourse (Table 3) (6). The remaining two indicators of healthy and responsible sexual behavior (both from NSFG) focused on approaches to reduce risk of HIV/STI and unintended pregnancy among sexually active adolescents and adults (15–44 years) (40). The first indicator assessed condom use: “sexually active unmarried persons who use condoms” (Table 2, 2.B) (6); the second indicator assessed contraceptive use more broadly: “percent of women aged 20–44 years at risk of unintended pregnancy who used most effective or moderately effective methods of contraception” (Table 2, 2.C) (6). In 2017–2019, during the last episode of vaginal sex, 22% of adolescent females used effective birth control and 67% of adolescent males used a condom and 62% of adolescent females (or their partners) at risk for unintended pregnancy used a most/moderately effective method of contraception (Table 3) (6).

Finally, we had particular difficulty identifying indicators of healthy, responsible, and respectful relationships in existing surveys and surveillance systems. Measures of relationships were rare and those we identified were focused on negative outcomes rather than aspects of positive relationships; therefore, we selected only one indicator for this topic: “people (>17 years) that report happiness with marriage” from the GSS (Table 2, 2.D) (45). Although limited to married persons only, it provided an initially useful measure of an important aspect of sexual health: relationship satisfaction. In 2021, 51% of married men and 49% of married women reported that they were very happy with their marriage (Table 3) (45).

### Objective 3. Increase use of high-quality, coordinated educational, clinical, and other preventive services that improve sexual health

The seven indicators selected for this objective assessed educational, clinical, and other preventive services for sexual health, including HIV/AIDS, STIs, viral hepatitis, and pregnancy. First, two indicators assess the percentage of schools teaching specific sexual health topics in a required course and having key violence prevention practices in place (Table 2, 3.A., 3.B.). In 2018, 18% of U.S. schools reported teaching 20 key sexual health topics to students in grade 6–8 and 43% in grades 9–12; such topics included modes of HIV/STI transmission and how to reduce the risk of HIV, STIs, and pregnancy, including the benefits of being sexually abstinent, negotiation and decision-making skills, and condom use (46). In 2018, 49% of secondary schools had policies in place to prevent bullying and sexual harassment (46). In addition, one indicator served as an overall measure of use of services: “sexually active persons (15–44 years) who receive reproductive health services” (Table 2, 3.C) (43). In 2011–2015, 78% of women received a reproductive-health service in the previous 12 months; however, only 13% of men reported the same (43). Two indicators were selected to measure the uptake of key recommendations for HIV and STI screening: (1) “people (>12 years) living with HIV who know their serostatus” (Table 2, 3.D) (6) and (2) “sexually active women (16–24 years) enrolled in Medicaid or commercial plans who are screened for chlamydia” (Table 2, 3.E) (6). In 2017, 86% of individuals living with HIV who were over the age of 12 years knew their serostatus (Table 3) (43). In 2018, the percentage of eligible women screened for chlamydia was 56% for those enrolled in Medicaid and commercial plans. For human papillomavirus (HPV), to prevent HPV-related cancers in men and women, “percent of adolescents aged 13–15 years received recommended doses of the HPV vaccine” (Table 2, 3.F) was selected as the indicator from the National Immunization Survey-Teen (NIS-Teen) (48). In 2020, 55% of adolescents received the recommended doses of the HPV vaccine (Table 3) (43). Additionally, “pregnant women (15–44 years) who receive early and adequate prenatal care” was chosen as an indicator of pregnancy care (Table 2, 3.G) from the National Vital Statistics System (NVSS) (49). In 2020, 77% of pregnant women received early and adequate prenatal services (50).

### Objective 4. Decrease adverse health outcomes, including HIV/STIs, viral hepatitis, unintended pregnancies, sexual violence, sexual dysfunction, and cancers in reproductive tracts

Given the traditional public health focus on adverse outcomes, we identified several indicators focusing on health outcomes. For HIV/AIDS, the indicator selected was “new HIV infections among adolescents and adults (>12 years)” (Table 2, 4.A) from the National HIV/AIDS Strategy (NHAS) (43). In 2017, there were an estimated 37,000 new HIV infections in the U.S. (Table 3) (51). The indicator selected for STIs was “syphilis in women aged 15–44 years and men who have sex with men (MSM)” (Table 2, 4.B) (43). Due to the variety of disease outcomes of concern for STI prevention, this indicator was selected as a complement to the service indicator (chlamydia screening). Of note, young women (ages 15–24) accounted for nearly half (45%) of the 1.7 million cases of chlamydia reported in 2017 (52). In 2017, the rate of primary and secondary syphilis (per 100,000) was 5.1 for women aged 15–44 year olds (Table 3) and 401.1 for MSM (6). Next, “new hepatitis B infections in adults (>17 years)” was selected as the indicator for viral hepatitis (Table 2, 4.C). In 2016, there were an estimated 20,900 new cases of hepatitis B infection in adults (Table 3) (43).

Three indicators were selected for pregnancy and sexual violence. For pregnancy, “pregnancies that are unintended (15–44 years)” and “pregnancy rates among adolescent (15–19 years) females” were selected as indicators (Table 2, 4.D, 4.E, respectively) (43). In 2011, 45% of pregnancies among females 15–44 years old were unintended (Table 3) (53). In 2016, 20.3 per 100,000 adolescents aged 15–19 gave birth in the United States (Table 3) (6). Finally, “prevalence of sexual violence in the past 12 months (>17 years)” was selected (Table 2, 4.F) from the National Intimate Partner and Sexual Violence Survey (NISVS) (54). In 2016–2017, 2.3% of females and 0.3% of males reported being raped in the previous 12 months (Table 3) (19). Other forms of unwanted sexual contact involving touch but not sexual penetration (e.g., being kissed in a sexual way or having sexual body parts fondled, groped, or grabbed) were more commonly reported −5.3% of females and 3.0% of males (Table 3).

A reoccurring measure of sexual dysfunction does not currently exist in the U.S. Therefore, at this point, we have not included an indicator here. To measure reproductive tract cancers, an indicator was selected from the North American Association of Central Cancer Registries (NAACCR), which monitors all cancer cases in the U.S. (21). This indicator measures the estimated annual number of: prostate, testis, and other penile cancer in men, and cervical, ovarian, uterine, vaginal, and vulvar cancer in women. In 2018, there were 110,070 new cases of genital system cancers among women and 176,320 among men (21).

## Discussion

For the past 20 years, there have been calls for a national health strategy and a more comprehensive sexual health approach to address the myriad of persistent sexual health problems in the U.S. Employing a sexual health approach requires shifting toward a holistic and integrated focus on health rather than disease. While strategies are being implemented by multisectoral stakeholders to support sexual health, reduce gender inequality, and combat stigma (e.g., HHS, The White House, CDC) (1, 6, 13, 15, 30), it is essential to establish a core set of national sexual health indicators that allow for measurement across time. The U.S. currently has an array of national surveys and surveillance systems with items related to sexual health. We argue that these indicators could be utilized to produce a baseline measure of national sexual health.

To be clear, we are not suggesting the indicators be used to form composite score. Rather, we envision these core sexual health indicators would be published in the form of an index that is publicly available and updated frequently. The Human Rights Campaign (HRC), for example, currently published an equality index “an annual comprehensive state-by-state report that provides a review of statewide laws and policies that affect LGBTQ+ people” (55). Similar to this HRC index (55) and other national sexual health indices (31–37), a U.S. sexual health scorecard could be used for: (1) monitoring purposes (e.g., to target areas for improvement); (2) research efforts (e.g., to examine associations with other structural drivers); (3) program design (e.g., to justify interventions); (4) policy guidance that entities such as CDC and HHS use to inform strategies and services to help promote sexual health in coming years.

To this end, we identified 23 indicators of sexual health that both highlight the current state of sexual health in the U.S. and also allow for future tracking. We were able to identify a set of indicators aligned with long-term sexual health objectives relevant to individuals, relationships, and communities. Each of these can be measured on a regular basis and used to monitor a breadth of issues relevant to sexual health. We conceive of these indicators as a baseline measure of the state of U.S. sexual health. It is our hope that these estimates will only improve in coming years and decades. The use and further refinement of these indicators is an essential aspect of resurgent national efforts to address sexual health (3, 11).

Our efforts were more successful for some objectives than others. Traditional public health measures—clinical services and adverse outcomes (Objectives 3 and 4)—tended to be collected frequently and consistently. This finding was not surprising given that surveillance of adverse outcomes is a primary, long-standing activity in U.S. public health systems. We found fewer measures of knowledge, communication (particularly with providers), attitudes, and behaviors and relationships related to sexual health (Objectives 1 and 2); and many of the measures that we did identify were limited in different ways. For example, in the absence of data on specific aspects of knowledge, we used exposure to education as an indirect measure; yet as a result, these indicators remain more focused on youth than young and older adults. Additionally, our measure of partner communication addresses a hypothetical situation rather than an actual event and is limited to adolescents and young adults. Finally, we were least successful in identifying measures of healthy relationships as our measure (i.e., “happiness in marriage”) is restricted to marriage and only an estimated 45% of women and 43% of men aged 15–44 years old in the U.S. are currently married (56). We were unable to identify a measure of sexual dysfunction (or its inverse, sexual function, or even sexual satisfaction), but we hope to do so in the future. One important national survey, the National Survey of Sexual Health and Behavior (NSSHB) could serve as a possible source for some of these measures if it were ongoing (57). Thus, it is our hope that proposed indicators will be reviewed and revised overtime to ensure usefulness—moving away a narrow focus on the specific (e.g., youth and marriage) toward a general focus on the life course.

Communication about sexual health with partners and parents was low: over 60% of youth would be embarrassed to discuss condom use with a new partner and roughly half of adolescents have talked to their parents/guardians about all four important sexual health topics. Formal sex education is limited, with roughly one-half of adolescents receiving it. Attitudes demonstrated the potential for discrimination, with around 36–40% of American adults agreeing that same-sex sexual relations between adults is wrong on some level. Stigmatizing attitudes toward homosexual populations may impact the sexual health of MSM, who are disproportionately impacted by HIV and other syndemics (14).

Additionally, findings for clinical and other preventive services were mixed. More females used reproductive-health services than men; however, 50-60% of young sexually active females did not receive recommended STI testing. Findings for sexual behaviors were also mixed. Less than 15% of youth aged 15 and under have had sexual intercourse. Only 67% of adolescent males used a condom at last sex. Finally, the burden of adverse outcomes remains high. Overall HIV incidence has been flat over the past 5 years but has increased substantially in young MSM (6, 43) while rates of STI such as gonorrhea remain far above Healthy People targets (6, 43). Although adolescent pregnancy has declined substantially over the past 20 years, the U.S. teen birth rate remains one of the highest of any industrialized country (58). Finally, for sexual violence, there are limited trend data, but current national estimates are cause for concern (19).

There are several limitations to our selected set of sexual health indicators. First, some indicators are imperfect matches to the constructs we want to measure. The use of education as an indicator for knowledge is a drawback. Measuring happiness in relationships should not be confined to marriage. Additionally, there is a lack of national data that address the sexual health of adults older than 49 years; thus, our indicators ignore the needs of older adults (8). We also found few quality measures of stigma-related to sexuality. The percentage of GSAs in schools taps into broader stigma but better measures are needed. Additional gaps in our set of indicators include limited available measures of communication (with providers and partners), attitudes, sexual dysfunction, sexual pleasure and satisfaction, and healthy relationships in national data sources. Finally, several of the selected indicators are not collected annually or biennially limiting frequent tracking of prevention efforts for some domains.

## Public health implications

As emphasized in the National Prevention Strategy, “healthy reproductive and sexual practices can play a critical role in enabling people to remain healthy and actively contribute to their community” (59). We identified a set of 23 sexual health indicators that allowed us to characterize the current state of sexual health in the U.S. as measured by breadth of issues related to sexual health knowledge, attitudes, service access and utilization, behaviors, relationships, and adverse health outcomes. These data are selected from an inventory of existing data systems that yield national estimates and allow for measurement across time. By tracking these indicators, we can measure progress made by larger national efforts, as well as multisectoral actors. There is potential for these indicators to have broad reaching impact beyond the STI epidemic or unintended pregnancy prevention—they could provide needed insight related to how social inequality is reproduced. For instance, these indicators can help us understand how syndemics encompassing HIV, viral hepatitis, substance use, maternal-child health, sexual violence, and reproductive health make it more difficult for some individuals and communities to protect their sexual health. Overall, our findings regarding the current state of sexual health in the U.S. provide broad evidence of sub-optimal sexual health in all domains measured, indicating the need for new approaches to address this important area of health and meet goals of national initiatives. Additionally, available indicators contain crucial gaps. Considerations for addressing these gaps include adding new measures (although we recognize the difficulty of doing this), creating research partnerships across disciplines, and developing a new comprehensive survey of sexual health as other countries have done (34–37).

## Data availability statement

The original contributions presented in the study are included in the article/supplementary material, further inquiries can be directed to the corresponding author.

## Author contributions

EC contributed to the framing and conceptualization of this article. JVF contributed to the framing, background, analysis, writing, and analysis as well as organizing the 2022 meeting. MBI contributed to the article's conceptualization, data review, literature, writing, organization of the paper, and also led 2010–2012 meetings. All authors contributed to the article and approved the submitted version.
